# The neutropenic diet and its impacts on clinical, nutritional, and lifestyle outcomes for people with cancer: a scoping review

**DOI:** 10.1017/jns.2024.61

**Published:** 2024-10-10

**Authors:** Trinity Gulliver, Melissa Hewett, Panagiotis Konstantopoulos, Lisa Tran, Evangeline Mantzioris

**Affiliations:** 1 Clinical and Health Sciences, University of South Australia, Adelaide, SA, Australia; 2 Department of Nutrition & Dietetics, Royal Adelaide Hospital, Adelaide, SA, Australia; 3 Alliance for Research in Exercise, Nutrition & Activity (ARENA), University of South Australia, Adelaide, SA, Australia

**Keywords:** Cancer, Infections, Neutropenic diet, Quality of life

## Abstract

The neutropenic diet (ND) is often recommended to people with cancer to reduce infection risk despite recommendations of clinical guidelines advising against its use. While recent literature suggests the ND does not reduce infection risk, other outcomes related to health, nutrition, and lifestyle are unknown. The aim of this review is to systematically scope the literature on the ND in people with cancer for all outcomes related to clinical health, nutrition, and lifestyle. Scientific databases were systematically searched. Eligible studies were in English, people with any cancer type, consuming an ND, any age group, date, or setting. Eligible study types were randomised control trials, observational studies, systematic reviews, and meta-analyses. Twenty-one studies met the inclusion criteria. Outcomes of interest found were infection rates, fever, mortality, antibiotic use, gastrointestinal side effects, comorbidities, biochemistry, hospitalisation, nutritional status, quality of life (QoL), well-being, and financial costs. Most research has focused on infection and mortality rates with few assessing hospitalisation rates, nutritional status, financial costs, and QoL. Most included studies found no significant differences between ND and comparator diet for mortality, antibiotics use, comorbidities, and QoL; however, several studies reported the ND significantly increased the risk of infection. Gaps in the literature included effect of ND on QoL in an adult population, microbiome, lifestyle changes, and financial burden. Further research is needed regarding how the ND affects the microbiome and QoL of its consumers, but in the interim, it is important for hospitals providing an ND to their patients to liberalise the ND wherever possible.

## Introduction

Haematological cancers originate in blood forming tissue, such as bone marrow or immune cells, and utilise chemotherapy as first line treatment followed by haematopoietic stem cell transplant (HSCT). HSCTs involve infusing stem cells from the bone marrow of the individual, taken prior to treatment (autologous) or from a matched donor (allogeneic). HSCT also involves a high dose of chemotherapy to destroy cancerous blood cells and suppress the immune system to allow the body to accept the stem cell transplant.^(1,2)^ This treatment may cause other healthy cells to be destroyed including neutrophils, which are integral in preventing infections.^(3)^ Due to the high dose of chemotherapy received, these individuals generally become neutropenic characterised by neutrophil counts below 1.5 × 10^9^ neutrophils per litre of blood, and reduction below 0.5 × 10^9^ neutrophils/l classified as severe neutropenia.^(4)^ The decline in neutrophil cell counts leads to an increased risk of infection, prolonged bleeding time due to low platelet count, increased pain, and reduced nutritional intake due to mucositis and tiredness.^(1)^

Due to the increased infection risk, individuals receiving cancer treatment in hospital in the 1960s would often be placed in sterile environments including laminar flow rooms and receive gut decontamination with antibiotics in addition to a ‘sterile’ diet.^(5)^ However, an early literature review in 1984 determined that protective environments did not reduce infection rates, but had many negative effects including poor psychological impact, increased costs, and increased staff labour associated with their use.^(6)^ Although complete protective environments are no longer employed, many healthcare institutions continue to provide patients with a neutropenic diet (ND),^(7)^ which have changed from very restrictive ‘sterile’ diets to one that limits ‘high-risk’ foods: raw fruits and vegetables, raw/undercooked meat, fish and eggs and unpasteurised dairy. Restriction of these foods is widely considered the basis of the ND; however, no set guidelines exist.^(7)^

In recent years the validity of the ND has been questioned. Numerous systematic reviews and meta-analyses^(8–14)^ have shown that the ND does not significantly reduce the risk of infection or mortality within this population. Additionally, The European Society for Clinical Nutrition and Metabolism (ESPEN), a major society in nutrition, do not recommend an ND.^(15,16)^ Despite this, clinicians^(17,18)^ and hospitals that perform HSCTs^(19–22)^ continue to provide patients with NDs in Switzerland,^(19)^ China,^(20)^ Italy,^(21)^ Germany,^(22)^ Austria,^(22)^ UK,^(17)^ and the US.^(18)^ All studies reported between 50 and 80% usage of the ND.^(17–22)^ Interestingly, the studies with >80% usage are the most recent studies within this area, published between 2018 and 2021.^(20–22)^

Although there are numerous studies that have assessed the ND and its impacts on infection and mortality rates, other outcomes aside from infection rates that are of importance in relation to diet during cancer have not been considered. To our knowledge, many of these additional areas have not been systematically reviewed and need to be considered as part of the overall impact of NDs in the health and lifestyle of people with cancer. Therefore, the aim of this research is to systematically scope the current evidence-base to identify studies on the ND and any outcomes for people with cancer that relate to their medical, nutritional, social, psychological, or physical health as well as costs associated with treatment. Additionally, gaps in the evidence and opportunities for future research will be identified.

## Methods

### Protocol

The methods for this scoping review were prospectively designed and registered with Open Science Framework on 19 July 2022 and can be accessed at https://osf.io/gan2p.

### Selection criteria

Included studies were required to be (i) in English, (ii) human studies assessing people with cancer of any type, (iii) consuming a ND as defined by the paper authors, (iv) any age group, date of study, or setting. There were minimal restrictions in the included studies in attempt to capture as many studies as possible. The eligible study types included randomised control trials (RCTs), retrospective, prospective, cohort, observational, comparative, systematic review, and meta-analyses.

Studies were excluded if (i) in a language other than English, (ii) non-human, (iii) assessed the wrong diet or (iv) outcomes were not related to the clinical health or lifestyle outcomes of the scoping review. Excluded study types included letters, conference proceedings, books, book chapters, and guidelines.

### Search strategy

The following databases were searched CINAHL Complete (EBSCO Publishing, Inc), The Cochrane Library (John Wiley & Sons, Ltd), Embase (Ovid), Emcare (Ovid), MEDLINE (Ovid), Scopus (Elsevier Science Publishers), and Web of Science (Clarivate Analytics).

The following search terms with Boolean operators were used in all databases with no other filters applied: “neutropenic diet*” or “low bacteria* diet*” or “low-bacteria* diet” or “low microbial diet*” or “low-microbial diet*” or “germ free diet*” or “germ-free diet*” or “sterile diet*”. Databases were searched from inception to 19th July 2022 with additional papers added from search alerts of the above searches in all databases between 20 July 2022 and 23 August 2023.

### Screening sources and data extraction

Screening was undertaken in Covidence (Veritas Health Innovation Ltd). Title and abstract screening were completed independently by two reviewers (TG + one of MH, PK, LT, EM) with any conflicts being resolved by discussion (TG + EM). Full text screening was completed independently by two authors (TG + one of MH, PK, LT, EM) with any conflicts being resolved by discussion (TG + EM) or by a third author if needed. Reasons for exclusion were given for each study at the full-text screening stage.

Data from included articles was extracted into Excel (Microsoft Corporation, Washington, US) using standardised tools formulated by one reviewer (TG) and checked by one reviewer (EM). Extraction was completed by one reviewer (TG) and checked by a second reviewer for accuracy (one of MH, PK, LT, or EM). The outcomes of interest that were extracted were infection rate, mortality rate, fever, antibiotic use, side effects (including diarrhoea, nausea, vomiting), comorbidities (including neutropenic enterocolitis, graft vs host disease, mucositis), hospitalisation, quality of life, diet acceptability, nutritional status, and costs. Data was extracted and used as it was presented in the corresponding paper, and the results are presented in a narrative summary.

### Critical appraisal

Critical appraisal was completed for all included studies using the most suitable JBI checklist for study type (https://jbi.global/critical-appraisal-tools). Appraisal was completed by TG and checked by EM. JBI critical appraisal checklist for descriptive/case series was used for all retrospective studies included in the review. This checklist is no longer listed on the JBI website but is however considered a key tool for critical appraisal of descriptive studies.^(23)^

## Results

**Fig. 1.**
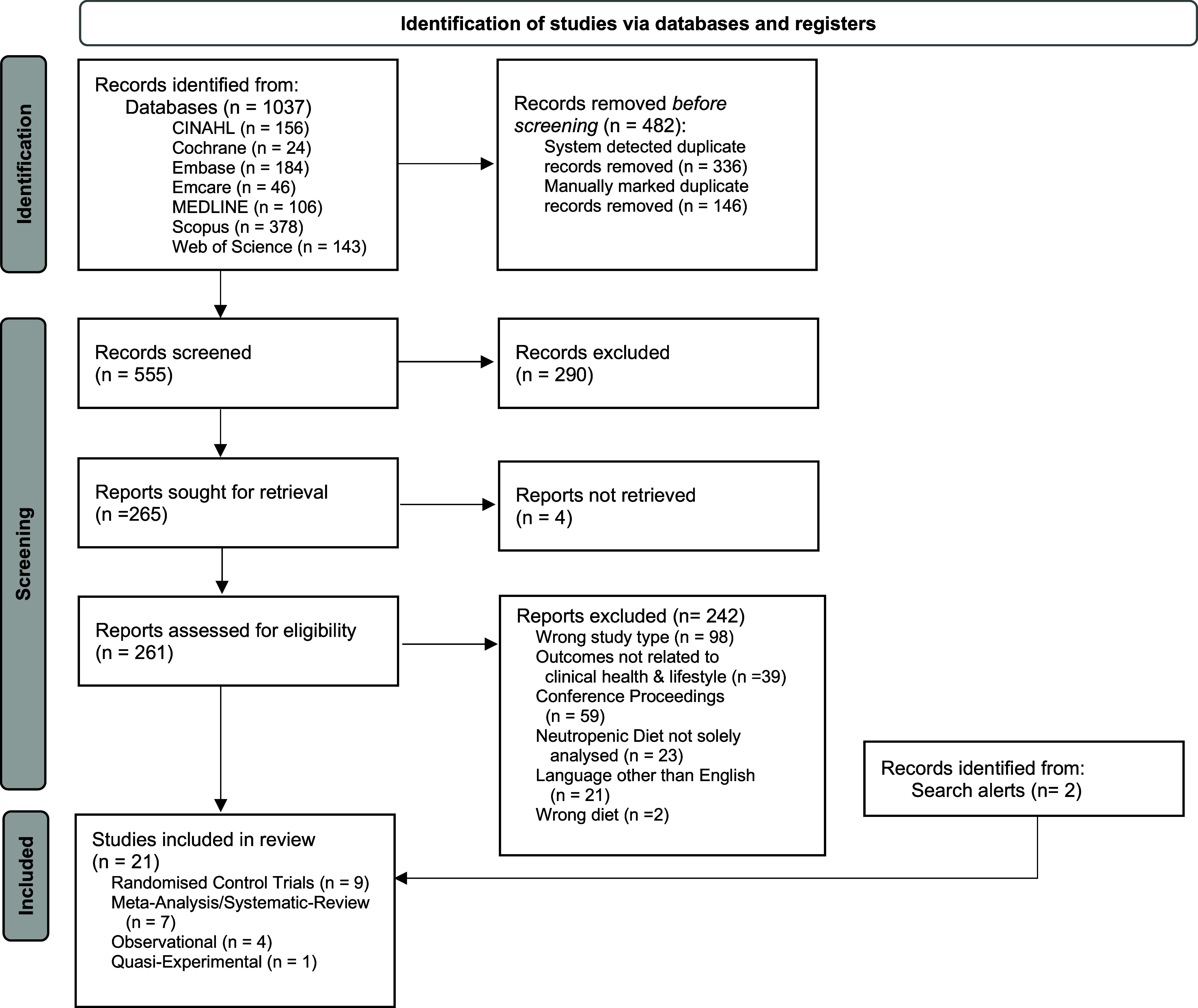
PRISMA Flow Diagram.

The initial search in July 2022 retrieved 1037 citations and after the removal of 481 duplicates, a total of 550 articles were available for title and abstract screening. Following initial screening, 264 articles were eligible for full-text screening. Following full-text screening 19 articles were deemed eligible for inclusion. Two articles were added from email alerts from the databases for a total of 21 included articles. Figure 1 shows the PRISMA Flow Diagram.

Nine studies were conducted in the United States,^(8,9,24–30)^ three in India,^(11,31,32)^ two in The Netherlands^(10,33)^ and Iran^(34,35)^ and one in Australia,^(36)^ China,^(12)^ Germany,^(37)^ The Philippines^(13)^ and Italy.^(14)^ Four studies^(24,30,32,35)^ included only paediatric participants and nine studies included only adult participants.^(14,25–28,33,34,36,37)^ Eight studies,^(8–13,29,31)^ including the seven systematic reviews/meta-analysis, included all age groups. Eleven studies were conducted in an inpatient hospital setting, and one in an outpatient setting.^(25)^ Two studies included both inpatients and outpatients^(24,29)^ and six of seven systematic reviews/meta-analyses^(8–13)^ included one or more of the outpatient studies whereas one^(14)^ only included studies with an inpatient population. All studies included participants with haematological cancers and four also included participants with oncological cancers^(24,25,29,32)^ however, none analysed results by cancer type. A summary of the characteristics of the included studies are shown in Table 1.

**Table 1.** Characteristics of Included Studies

Study (country)	Design (setting)	Participants		Diet specifications		Critical appraisal
		Number	Age	Neutropenic	Control	
DeMille 2006(25) (United States)	Prospective (Outpatient)	ND: 16 CD: 7	18–70	American Dietetic Association food safety guidelines in addition to restricted foods: – Fresh and dried fruit – Raw vegetables – Unpasteurised juice – Raw/undercooked meat, fish and eggs – Dried and raw herbs – Buffet and street food	Those non-compliant with neutropenic diet.	6/9
Trifilio 2012(27) (United States)	Retrospective (Inpatient)	ND: 363 CD: 363	ND: 18–78 CD: 18–76	Diet used during neutropenic period only with restricted foods: – Fresh fruit – Raw vegetables – Black pepper – Raw/undercooked meats and cheeses – Cold smoked fish – Raw/unpasteurised dairy – Raw miso, grains and seeds – Brewer’s yeast	Academy of Nutrition and Dietetics approved hospital diet with restricted foods: – Raw tomatoes – Raw/undercooked meats and cheeses – Cold smoked fish – Raw/unpasteurised dairy – Raw miso, grains and seeds – Brewer’s yeast	6/9
Taggart 2019(30) (United States)	Prospective (Inpatient)	ND: 49 CD: 53	ND: 11.7 CD: 9.2	Restricted Foods: – Fresh fruit – Raw vegetables – Unpasteurised dairy – Raw/undercooked eggs, meat and fish – Raw blue cheese – Dairy from bulk machines – Deli meats – Raw grains, yeast and honey – Buffet meals – Fast food (if not freshly prepared) – Sharing food	Modified Bone Marrow Transplant Diet Restricted Foods: – Unpasteurised dairy – Raw blue cheese – Dairy from bulk machines – Undercooked meat and fish	8/9
Heng 2020(36) (Australia)	Retrospective (Inpatient)	ND: 79 CD: 75	ND: 50–63 CD: 53–69	Hospital food safety guidelines in addition to restricted foods: – Fresh fruits – Raw vegetables – Undercooked meat and eggs – Cold meats – Yoghurt – Cold desserts – Fresh herbs – Black pepper (added after cooking)	Hospital food safety guidelines consisting of restricted foods: – Undercooked meat and eggs	6/9
Jakob 2021(37) (Germany)	Retrospective (Inpatient)	ND: 1043 CD: 1043	ND: 40–63 CD: 37–61	Restricted Foods: – Fresh fruits – Raw vegetables – Raw grains and nuts – Raw dairy and eggs – Tap water	Hospital food safety guidelines consisting of restricted foods: – Unpasteurised dairy – Raw/undercooked eggs, poultry, fish, and meat	7/9
Moody 2006(24) (United States)	RCT (Inpatient + Outpatient)	ND: 9 CD: 10	ND: 4.4 CD: 4.1	FDA restrictions in addition to restricted foods: – Fresh fruit (excluding fruits able to be hand peeled) – Raw vegetables – Aged cheeses – Cold cuts – Fast foods – Takeaway	FDA Food Safety Guidelines consisting of restricted foods: – Unpasteurised milk, cheese, juice – Raw/undercooked eggs, poultry, fish, and meat	11/13
van Tiel 2007(33) (Netherlands)	RCT (Inpatient)	ND: 10 CD: 10	ND: 40–69 CD: 30–68	Restricted foods: – Raw vegetables – Soft cheeses – Raw meats – Most fresh fruits – Tap water – Raw spices Use of single serve containers for food	Normal hospital diet. Did not specify dietary restrictions.	9/13
Gardner 2008(26) (United States)	RCT (Inpatient)	ND: 75 CD: 78 CDN: 53	ND: 17–88 CD: 47–84 CDN: 49–81	Restricted foods: – Fresh fruit – Raw vegetables	Fresh fruits and vegetables not restricted. Did not specify dietary restrictions.	8/13
Lassiter 2015(28) (United States)	RCT (Inpatient)	ND: 25 CD: 21	ND: 45 CD: 45	FDA Food safety guidelines. Allowed to consume cooked foods and thick-skinned fruits	FDA Food safety guidelines with no further food restrictions	10/13
Moody 2017(29) (United States)	RCT (Inpatient + Outpatient)	ND: 77 CD: 73	ND: 12 CD: 11	FDA restrictions in addition to restricted foods: – Raw vegetables – Fresh fruit (excluding fruits able to be hand peeled) – Aged cheeses – Cold cuts – Raw nuts and nut butters – Yoghurt – Unpasteurised dairy/fruit juice – Undercooked food – Fast foods and Takeaway	FDA Food Safety Guidelines consisting of restricted foods: – Unpasteurised milk, cheese, juice – Raw/undercooked eggs, poultry, fish, and meat	11/13
Jalali 2018(34) (Iran)	RCT (Inpatient)	ND: 25 CD: 25	ND: 40 CD: 41	Restricted Foods: – Fresh fruit (excluding fruits able to be hand peeled) – Water not boiled – Unpasteurised dairy – Raw/undercooked meat and eggs	Mediterranean Neutropenic Diet – Consumed 30mL olive oil daily – Same restrictions as ND group	9/13
Hosseini 2020(35) (Iran)	RCT (Inpatient)	ND: 25 CD: 25	ND: 40.80 CD: 38.36	Restricted Foods: – Raw fruits (excluding fruits able to be hand peeled) – Raw vegetables – Raw/undercooked eggs and meat – Unpasteurised dairy – Un-boiled water	Neutropenic Diet + Vitamin C (500 mg tablet daily) Restricted Foods: – Raw fruits (excluding fruits able to be hand peeled) – Raw vegetables – Raw/undercooked eggs and meat – Unpasteurised dairy – Un-boiled water	7/13
Gupta 2022(32) (India)	RCT (Inpatient)	ND: 21 CD: 21	ND: 3–13 CD: 3–12	Hospital food safety guidelines in addition to restricted foods: – All raw foods	Hospital food safety guidelines	11/13
Radhakrishnan 2022(31) (India)	RCT	ND: 102 CD: 98	ND: 1–60 CD: 1–59	American Cancer Society Food safety guidelines in addition to restricted foods: – Fresh fruit – Unpasteurised fruit juice – Raw vegetables	American Cancer Society food safety guidelines	10/13

Study (Country)	Design	Included Studies	Inclusion Criteria	Exclusion Criteria	Critical Appraisal
van Dalen 2012(10) (Netherlands)	Systematic Review	3	– Randomised Control Trials – Comparing neutropenic diet to control – Cancer patients receiving chemotherapy causing neutropenia – Adult and paediatric population	– Children under 1 year of age	9/11
Ball 2019(9) (United States)	Meta-Analysis	5	– Randomised Control Trial – Compares liberal diet to a neutropenic diet – Assessed rates of infection	– Did not report outcomes relating to neutropenic diet compared to unrestricted diet	5/11
Sonbol 2019(8) (United States)	Systematic Review/Meta-Analysis	6	– Neutropenic diet compared to regular diet in neutropenic patients with cancer Adult and paediatric populations – Comparative, retrospective, prospective studies	– Single-arm trials	10/11
Ramamoorthy 2020(11) (United States/India)	Systematic Review	11	– Infection rates: bacteraemia, pneumonia, fungaemia – Infection related mortality and all-cause mortality	NA	4/11
Ma 2022(12) (China)	Systematic Review/Meta-Analysis	6	– Diagnosed with leukaemia and neutropenic – Randomised Control Trial only – Neutropenic diet intervention – Standardised diet control	– Duplicates – Letters and abstracts	8/11
Ng 2022(13) (Philippines)	Meta-Analysis	3	– Paediatric population – Undergoing chemotherapy – Randomised Control Trial only – No limits on treatment phase – No language limits	– Adult population – All other study types	7/11
Matteucci 2023(14) (Italy)	Systematic Review	12	– Adult population – Haematological malignancy – Meta-analyses and observational studies – Published from May 2015 to January 2023	– Paediatric population – All other study types – Published prior to May 2015	11/11

ND, neutropenic diet; CD, comparator diet; RCT, randomised control trial; CDN, comparator diet not randomly assigned, ages presented as mean ages or age range.

There are no guidelines for the use of the ND and the diet can vary between hospitals in the foods restricted. This is reflected in the differences between the NDs in the included studies. All included studies restricted most raw fruits and vegetables – some allowed fruits and vegetables with thick skin or that could be hand-peeled.^(24,28,29,35)^ Other restricted foods included raw grains,^(27,30,37)^ raw nuts/nut butters,^(29,37)^ raw seeds,^(27)^ raw miso,^(27)^ yoghurt,^(29,36)^ dairy from bulk machines (i.e. soft serve ice cream, frozen yoghurt), raw honey,^(30)^ dried/raw herbs and spices,^(25,27,33,36)^ yeast,^(27,30)^ cold desserts^(36)^ tap/un-boiled water,^(33–35,37)^ takeaway/fast food,^(24,29,30)^ buffet/street food,^(25,30)^ and sharing food.^(30)^

The major comparator diet in the included studies was the food safety diet, used by nine studies.^(24,27–32,36,37)^ This diet restricts unpasteurised eggs, dairy, raw/undercooked meat and fish and has regulations on food handling, washing, preparation, and storage.^(38)^ One study termed the comparator diet ‘Modified Bone Marrow Transplant Diet’ however restrictions were similar to the food safety diet.^(30)^ Two studies^(27,33)^ used a standard hospital diet which followed the food safety diet with some additional restrictions: raw tomatoes, cold smoked fish, raw miso, raw grains/seeds, and brewer’s yeast. One study did not impose any restrictions for the comparator diet,^(26)^ and another included participants in the comparator group if they were non-compliant with the ND.^(25)^

The incidence of infections was a major outcome in all but two of the 21 included studies. However, the type of infections, grouping of infection types, and the method of reporting varied amongst studies. As shown in Table 2, we report these results as they have been reported in the included studies.

**Table 2.** Effect of the Neutropenic Diet on Rates of Infection

Study	Total infection	Total infection + fever	Fever	Major infection	Minor infection	Gut colonisation	Pneumonia	Bacteraemia	Fungaemia	Bacteraemia + fungaemia	Viral infection
DeMille 2006^(25)^	−	NA	NA	NA	NA	NA	NA	NA	NA	NA	NA
Trifilio 2012^(27)^	−^a^	NA	NA	NA	NA	NA	NA	NA	NA	NA	NA
	↑^b^										
Taggart 2019^(30)^	NA	NA	NA	NA	NA	NA	NA	−	NA	NA	−
Heng 2020^(36)^	−	NA	−^f^	NA	NA	NA	NA	−	NA	NA	NA
			↑^g^								
Jakob 2021^(37)^	−	NA	−	NA	NA	NA	NA	−	NA	NA	NA
Moody 2006^(24)^	−	NA	NA	NA	NA	NA	NA	NA	NA	NA	NA
van Tiel 2007^(33)^	NA	NA	−	NA	NA	−	NA	−	−	NA	NA
Gardner 2008^(26)^	−	−	−	−	−	NA	−	NA	NA	↓	NA
Lassiter 2015^(28)^	NA	NA	NA	NA	NA	NA	NA	−	NA	NA	NA
Moody 2017^(29)^	−	NA	−	NA	NA	NA	NA	NA	NA	NA	NA
Gupta 2022^(32)^	↑	NA	−	NA	NA	NA	−	NA	NA	NA	NA
Radhakrishnan 2022^(31)^	−	NA	−	−	−	↓^j^	−	−	NA	NA	NA
						−^k^					
van Dalen 2012^(10)^	NA	−	NA	NA	NA	NA	NA	NA	NA	NA	NA
Ball 2019^(9)^	−	NA	NA	NA	NA	NA	NA	NA	NA	NA	NA
Sonbol 2019^(8)^	NA	↑^d^	NA	↑^h^	NA	NA	NA	NA	NA	−	NA
		−^e^		−^i^							
Ramamoorthy 2020^(11)^	−	NA	NA	NA	NA	NA	NA	NA	NA	NA	NA
Ma 2022^(12)^	−	NA	NA	NA	NA	NA	NA	NA	NA	NA	NA
Ng 2022^(13)^	NA	NA	−	NA	NA	NA	NA	−	NA	NA	NA
Matteucci 2023^(14)^	−^c^	NA	NA	NA	NA	NA	NA	NA	NA	NA	NA

NA, Not Assessed.

a During neutropenia.

b Overall and when neutropenia had resolved.

c Significant differences not reported however of the six studies 4/6 found no significant difference 1/6 did not include a comparator and 1/6 ND increased infection risk for HSCT transplant recipients only.

d HSCT recipients and overall.

e Non-HSCT recipients.

f Cases of febrile neutropenia.

g Number of febrile days.

h HSCT recipients.

i Non-HSCT recipients and overall.

j At baseline.

k Day 15 of study.

− No significant difference between Neutropenic Diet and comparator, ↑Neutropenic Diet increases risk, ↓Neutropenic Diet decreases risk.

Total infection, reported in 13 studies^(9,11,12,14,24–27,29,31,32,36,37)^ included infection of any body-site with any bacteria, fungi, or virus. One RCT^(32)^ reported a significantly higher rate of infection in the ND group with 12 infections (n = 21) compared to nine in the comparator group (n = 21) (P = 0.049).^(32)^ A retrospective study^(27)^ found a significant difference between the diets for total diagnostically confirmed infections – 135 infections in the ND group compared to 106 in the comparator group (P = 0.03). Infections present when neutropenia had resolved was also significantly higher in the ND group compared to the comparator group (P = 0.01). However, infection during neutropenia was not significantly different (P = 0.22).^(27)^ Ten of the 13 studies which reported on total infection did not find any significant difference between diet groups. Overall, from the included studies the ND either results in no significant difference in infection rates compared to the comparator diet (n = 12 studies) or increases the infection rate (n = 2 studies).

A further three studies reported combined rates of total infection and/or fever.^(8,10,26)^ A systematic review/meta-analysis^(8)^ found higher rates of infection in the ND group for the total population (RR 1.17, 95% CI (1.04–1.32)). When haematopoietic stem cell transplant (HSCT) recipients following the ND were compared to those following the comparator diet, significantly higher rates of infection in the ND group were demonstrated (RR 1.25, 95% CI (1.02–1.54)). However, no significant difference was seen between diet groups for participants who were not recipients of HSCT.^(8)^

Major infection was assessed in three studies;^(8,26,31)^ however, as there is no clinical definition of what constitutes a major infection, there were differences in the definition between studies. All three studies included pneumonia, bacteraemia, or fungaemia as major infections,^(8,26,31)^ whilst one study also included urinary tract infection, meningitis, cellulitis, or diarrhoea.^(31)^ A systematic review/meta-analysis reported a significantly higher rate of major infection in the ND group when only assessing participants who were HSCT recipients (RR 1.25, 95% CI (1.02–1.54)). No significant difference was seen in participants who were not transplant recipients or when all participants were assessed together.^(8)^

Minor infection was assessed by two studies;^(26,31)^ however, as there is no clinical definition of what constitutes a minor infection, both studies included all other infections not defined as a major infection. Both studies found no significant difference between diet groups.^(26,31)^

Gut colonisation by pathogenic bacteria or yeasts was assessed in two RCTs by faecal analysis.^(31,33)^ Radhakrishnan, Lagudu^(31)^ reported significantly higher rates of colonisation in the comparator group (n = 40/96) compared to the ND group (n = 29/102) at baseline (P = 0.05). Bacteria isolated from stool included multidrug resistant (MDR) *Escherichia coli*, MDR *E. faecalis*, MDR *Klebsiella pneumoniae*, MDR *E. faecium*, Vancomycin resistant *E. faecium*. A positive stool culture at baseline is considered more likely to be reflective of the diet consumed while participants are in the community. At the end of the study (day 15), there were no differences, indicating that the diet did not significantly affect gut colonisation.^(31)^ No significant differences were reported in the study by van Tiel, Harbers.^(33)^

Pneumonia is the inflammation of the lungs caused by bacteria, fungi, or viruses^(39)^ and while there is no evidence linking it to diet, it was assessed in three studies.^(26,31,32)^ Two studies found no significant difference between diet groups^(26,31)^ and the third did not analyse statistical significance, however they reported two pneumonia cases in the ND group (n = 21) and one in the comparator group (n = 21).^(32)^

Bacteraemia, presence of bacteria in the bloodstream was assessed in seven studies, none of which found a significant difference between diet groups.^(13,28,30,31,33,36,37)^ *Fungaemia, the presence of fungi in the bloodstream was assessed by o*ne RCT which found no significant difference between diet groups.^(33)^ Combined bacteraemia or fungaemia was assessed in two studies,^(8,26)^ a quasi-experimental study found significantly higher rates in the comparator group (n = 17) than the ND (n = 7) (P = 0.03);^(26)^ however, a systematic review/meta-analysis found no significant differences between diet groups.^(8)^

Norovirus, which causes gastrointestinal infection^(40)^ was assessed in an observational study which reported no significant differences between diet groups.^(30)^

Neutropenic fever or febrile neutropenia is characterised as a high temperature (>38.3°C or >38°C on two occasions).^(41)^ Eight studies assessed fever with five reporting on neutropenic fever,^(13,24,31,32,36)^ four reported on fever of unknown origin (>38.3°C on multiple occasions for three weeks, with diagnosis unclear after one week^(42)^),^(26,31,36,37)^ one assessed persistent fever^(37)^ and one assessed high or low temperature,^(33)^ with none of the eight studies reporting significant differences between diet groups.

Antibiotic use was assessed in two studies with different metrics; length of antibiotic use (the consecutive number of days which an antibiotic is used)^(32,33)^ and antibiotic duration (the total amount of time in which antibiotics are used).^(32)^ Neither study found a significant difference between diet groups for use of antibiotics (see Table 3).

**Table 3.** Effect of the Neutropenic Diet on Clinical Factors

Study	Mortality		Antibiotic use		Gastrointestinal			Comorbidities		
	Actual	Probability	Length	Duration	Diarrhoea	Nausea	Vomiting	NE	GvHD	Mucositis
Trifilio 2012^(27)^	−	NA	NA	NA	−	NA	NA	NA	−	NA
Taggart 2019^(30)^	NA	NA	NA	NA	NA	NA	NA	NA	−	NA
Heng 2020^(36)^	−	NA	NA	NA	−	NA	NA	−	NA	NA
Jakob 2021^(37)^	NA	−	NA	NA	↑	↑	NA	NA	NA	NA
Moody 2006^(24)^	NA	NA	NA	NA	−	NA	−	NA	NA	NA
van Tiel 2007^(33)^	NA	NA	−	NA	NA	NA	NA	NA	NA	NA
Gardner 2008^(26)^	NA	−	NA	NA	NA	NA	NA	NA	NA	NA
Moody 2017^(29)^	NA	NA	NA	NA	NA	NA	NA	NA	NA	−
Gupta 2022^(32)^	−	NA	−	−	NA	NA	NA	NA	NA	NA
Radhakrishnan 2022^(31)^	−	NA	NA	NA	NA	NA	NA	NA	NA	NA
Sonbol 2019^(8)^	−	NA	NA	NA	NA	NA	NA	NA	NA	NA
Ramamoorthy 2020^(11)^	−	NA	NA	NA	NA	NA	NA	NA	NA	NA
Ma 2022^(12)^	−	NA	NA	NA	NA	NA	NA	NA	NA	NA
Matteucci 2023^(14)^	−^a^	NA	NA	NA	↑^b^	↑^b^	NA	NA	NA	NA

NA, not assessed; NE, neutropenic enterocolitis.

a Significant difference not reported 3/3 studies included had no significant differences between groups.

b Only included results from Jakob, Classen.^(37)^

− No significant difference between Neutropenic Diet and comparator, ↑Neutropenic Diet increases risk.

Gastrointestinal side effects such as diarrhoea, nausea, and vomiting during cancer treatment may be caused by chemotherapy,^(43)^ use of antibiotics which causes healthy bacteria of the gut to be destroyed and/or, use of other medications such as opioids.^(43)^ These symptoms are also commonly attributed to *Clostridium difficile* infection.^(44)^ Diarrhoea was assessed in five studies^(14,24,27,36,37)^ and only one study found a significantly higher rate in the ND group (P < 0.001). The remaining studies saw no significant differences. Two studies reported on *Clostridium difficile* – with both reporting no significant difference between groups – however, no link was made between the incidence of diarrhoea and *Clostridium difficile* infection.^(27,36)^ Nausea was assessed in two studies, a retrospective study reported a significantly higher rate of nausea in the ND group^(37)^ and a systematic review included only findings from the retrospective study.^(14)^ Vomiting was assessed in one study which reported two of nine participants in the ND group and two of ten in the comparator diet group had instances of vomiting, however, significance was not reported^(24)^ (see Table 3).

Neutropenic enterocolitis is the inflammation of the gastrointestinal tract occurring in a neutropenic individual. Two studies^(32,36)^ assessed neutropenic enterocolitis, one study reported higher rates in the ND group (P = 0.044) and the other reported no significant difference between groups.^(36)^

Graft vs Host Disease (GvHD) can occur post-stem cell transplant when donor T-cells attack healthy cells of the recipient. Two observational studies assessed GvHD;^(27,30)^ however, neither saw a significant difference between diet groups.

Mucositis, defined as, inflammation of the mouth and/or gut, was assessed by one RCT^(29)^ which reported four cases in the ND group and two in the comparator group, however this was not significant.

Mortality was assessed in ten studies;^(8,11,12,14,26,27,31,32,36,37)^ however, no studies reported any significant differences between groups (see Table 3).

Serum albumin, whilst no longer used as a sole indicator of nutritional status, recent literature suggests decreased levels may be associated with gut dysbiosis.^(45)^ Two RCTs^(34,35)^ assessed serum albumin levels and both found significantly lower levels in ND groups compared to the comparator diets post-intervention (see Table 4). This may indicate a decreased dietary intake or gut dysbiosis. Neither study reported on fluid status, inflammation, microbiome, or other GI related side effects associated with decreased serum albumin levels.

**Table 4.** Effect of the Neutropenic Diet on Biochemical Factors, Hospitalisation, Nutritional Status, and Well-being

Study	Biochemical	Hospitalisation		Nutritional status		Wellbeing		
	Serum albumin	Length of Stay	Admissions	Weight change	PG-SGA	Quality of life	Diet acceptability	Diet adherence
DeMille 2006^(25)^	NA	NA	−	NA	NA	NA	NA	NA
Trifilio 2012^(27)^	NA	−	NA	NA	NA	NA	NA	NA
Taggart 2019^(30)^	NA	NA	NA	NA	NA	NA	−	NA
Heng 2020^(36)^	NA	−	−	NA	NA	NA	NA	NA
Jakob 2021^(37)^	NA	NA	NA	↑^c^	NA	NA	NA	NA
				−^d^	NA	NA	NA	NA
Moody 2006^(24)^	NA	NA	NA	NA	NA	↑^e^	−	SNR
						−^f^	NA	NA
Lassiter 2015^(28)^	NA	NA	NA	NA	NA	−	NA	NA
Moody 2017^(29)^	NA	NA	NA	NA	NA	−	NA	NA
Jalali 2018^(34)^	↑^a^	NA	NA	−	↑	NA	NA	NA
	−^b^							
Hosseini 2020^(35)^	↑^a^	NA	NA	−	↑	NA	NA	NA
	−^b^							
Gupta 2022^(32)^	NA	−	−	NA	NA	NA	NA	−
van Dalen 2012^(10)^	NA	NA	NA	NA	NA	NA	−	NA
Matteucci 2023^(14)^	NA	NA	NA	↑^c,^ ^g^	NA	NA	NA	NA
				−^d,^ ^g^				

NA, Not Assessed; SNR, Significance Not Reported.

a Neutropenic Diet significantly decreases serum albumin when compared to diet with additional Vitamin C or olive oil and when compared pre and post intervention.

b Neutropenic Diet is not significantly different from diet with additional Vitamin C or olive oil pre intervention and diet with additional Vitamin C olive oil is not significantly different pre and post intervention.

c Neutropenic diet significantly increases risk of weight loss of between 1 and 3 kg.

d No significant difference between diets for weight loss of greater than 3 kg.

e Neutropenic diet significantly lower QoL score for PEDS core module.

f No significant difference between diet groups for PEDS cancer module.

g only included results from Jakob, Classen.^(37)^

↑Neutropenic Diet increases risk, − No significant difference between Neutropenic Diet and comparator.

Four studies assessed hospitalisation by length of stay^(27,32,36)^ or admissions.^(25,32,36)^ A retrospective study^(27)^ reported that those in the ND group (n = 363) who underwent HSCT spent, on average, one day longer in hospital than those following the comparator diet (n = 363), but level of significance was not reported.^(27)^ No significant difference was seen in the remaining two studies.^(32,36)^ None of the three studies which assessed hospital admissions found a significant difference between diet groups^(25,32,36)^ (see Table 4).

Patient Generated Subjective Global Assessment (PG-SGA) is a common, validated tool used in cancer patients to evaluate nutritional status.^(46)^ The PG-SGA categorises individuals into three groups: ‘appropriate nutrition’, ‘prone to malnutrition’ or ‘severe malnutrition’. Two RCTs^(34,35)^ reported a significantly higher proportion of the ND group being placed into the ‘prone to malnutrition’ or ‘severe malnutrition’ categories.

Weight change was assessed in four studies.^(14,34,35,37)^ A retrospective study reported a significantly greater proportion of those in the ND group losing between 1 and 3 kg (P = 0.05), however further sub-analysis showed no significance for weight loss greater than 3 kg.^(37)^ This study only reported absolute weight loss and did not report percentage weight loss, hence making it difficult to determine if this weight loss is clinically significant. Weight loss was an outcome in the systematic review by Matteucci, De Pasquale^(14)^ and the only included study for this outcome was Jakob, Classen.^(37)^ As no further analysis was conducted in the systematic review, results were the same.^(14)^

One study published in 2007^(33)^ assessed the total financial costs as a secondary outcome of the ND contrasted with the comparator diet at different stages of care – including hospital costs, other healthcare costs, and inability to work. The areas in which costs are associated were identified, however the reason for cost differences were not determined. Whilst they reported higher costs for the comparator diet during hospitalisation, in contrast during follow-up and in total, the ND had higher costs, however significance of these results was not reported.^(33)^

Quality of Life (QoL) is defined by the World Health Organisation as ‘an individual’s perception of their position in life in the context of the culture and value systems in which they live and in relation to their goals, expectations, standards, and concerns’.^(47)^ Two RCTs from the same research group^(24,29)^ assessed QoL in paediatric cancer patients (Table 4) using the Paediatric Quality of Life Inventory. These two studies were similar in design and in the outcomes assessed, however the 2017 study^(29)^ had more participants (ND = 77, CD = 73) compared to the 2006 study (24) (ND = 9, CD = 10). In the 2006 study, they found the Core QoL of the ND group was significantly lower (indicating lower overall QoL) than the comparator group (P < 0.05), however, this was not significant for cancer specific QoL.^(24)^ In the 2017 study, they did not find a significant difference between diet groups.^(29)^

Diet acceptability was assessed by three different metrics across three studies^(10,24,30)^; ease of following assigned diet, food not tasting how participants remembered, and inability to consume desired foods. All three RCTs found no significant difference between ND and comparator diet groups for diet acceptability.^(10,24,30)^

Diet adherence was measured by two different metrics in two RCTs; number of meals for which participants (n = 19) were following their assigned diet^(24)^ and number of weeks participants (n = 42) were following the assigned diet.^(32)^ Both studies found significantly better adherence in the comparator groups. Participants were reported to have adhered to the ND (n = 9) 94.10% of the time and to the comparator diet (n = 10) 99.99% of the time.^(24)^ Participants followed the ND for 93 of the 98 weeks (n = 21) and the comparator 94 of the 98 weeks (n = 21), however, this was not significantly different.^(32)^

## Discussion

The aim of the present scoping review was to systematically search the literature and identify articles which assessed the use of the ND for those undergoing cancer treatment on any outcomes relating to medical, nutritional, social, psychological, physical health, and all associated costs. We identified 21 relevant articles which covered outcomes including participants’ clinical health with most assessing infection and mortality rates. Ten of sixteen outcomes included in the present study have not previously been included in systematic reviews.

Clinical outcomes identified in this scoping review were related to risk of infection, mortality, and fever in 19 of the 21 studies. The majority of studies observed no significant difference between groups, which aligns with the rationale for recommendations of the clinical guidelines for nutrition in cancer patients from The European Society for Clinical Nutrition and Metabolism (ESPEN) published in 2016^(15)^ and updated in 2021.^(16)^ Based on the evidence base at the time, ESPEN recommended that “There are insufficient consistent clinical data to recommend a low bacterial diet for patients more than 30 days after allogeneic transplant”.^(15,16)^ As such the ESPEN guideline for hospital nutrition.^(48)^ was “Neutropenic diets (also called ‘germ-free’, ‘no microbial’ or ‘sterilised’ diets) shall not be used (e.g. in neutropenic patients with cancer including haematopoietic stem cell transplant patients)”,^(48)^ it received an A grade recommendation as it was supported by a strong evidence base, including a meta-analysis^(8)^ and a Cochrane review.^(10)^ The recommendation received strong consensus from ESPEN members, and it was recommended that this population follow food safety guidelines.^(48)^

The ND generally includes well-cooked meat and excludes raw fruit and juices, however, within a population of Haematopoietic Stem Cell Transplant (HSCT) recipients the main food aversions were shown to be meat, specifically beef and chicken, due to the association with dysphagia.^(49)^ Preferred foods included fruit, fruit juices, and soup due to the association with improved gastrointestinal symptoms i.e. nausea.^(49)^ The ND has been shown to have reduced Vitamin C^(50,51)^ and fibre^(50)^ due to restrictions on raw fruits and vegetables, which increases the risk of nutrient deficiencies in this population.^(50,51)^ These nutrient deficiencies coupled with a decreased overall energy intake could lead to malnutrition. Malnutrition associated with cancer is common, with rates of 30–40% in Australia and was an outcome in two included studies.^(34,35)^ The combination of loss of taste and gastrointestinal symptoms, such as nausea could lead to decreased intake of food which may result in malnutrition.^(49)^

Weight loss during cancer treatment may be caused by a decreased intake of food due to loss of taste and other common side effects from treatment such as nausea and vomiting;^(49)^ however, this scoping review determined that weight loss was seen in people with cancer following an ND.^(34,37)^ Low weight in people with cancer is concerning as it has been shown to decrease overall survivorship in allogeneic HSCT recipients^(52)^ and may be a sign of cancer cachexia, characterised by lower skeletal muscle mass. Moreover, cachexia requires medication, nutrition therapy, exercise, and psychosocial interventions and if untreated can reduce positive chemotherapy outcomes, increase side effects, and decrease survivorship.^(53)^

The impact on the microbiome and associated health problems have been identified in this scoping review as one of the major gaps in ND research. The understanding of the importance of the microbiome is developing, and it can affect cancer development, prevention, and treatment efficacy, conversely, treatment can also affect the microbiome.^(54)^ Problematically, chemotherapy can cause gut dysbiosis – loss of diversity or changes to the gut microbiota, which affects the immune system and increases infection risk.^(55)^ Antibiotics, commonly prescribed during cancer treatment, alter the balance of bacteria in the gut and negatively impact immunotherapy.^(55)^ One study found that multi-drug resistant bacteria were detected in the faeces of ND patients in greater quantities than those following the comparator diet at baseline.^(31)^ Interestingly, the participants included only those receiving induction chemotherapy, therefore, they would not have begun consuming their study diet, and it was not specified whether they had any prior use of antibiotics. Participants’ diets prior to the study were not assessed. No studies have been conducted to demonstrate the impact of the ND on the gut microbiome, however the ND has been shown to have reduced fibre when compared to a standard hospital diet.^(50)^ As raw fruits and vegetables are limited in the ND, this may limit the number of sources of fibre and probiotics. This may have an impact on the microbiome but as no research has been conducted this remains unknown.^(50)^

Evaluation of differences in financial costs between the ND and the comparator diet was only assessed in one study. Overall, the ND had higher costs (hospital costs, other healthcare costs and, inability to work) of EU€1,760 more compared to a standard hospital diet for the duration of treatment (EU€41,769 vs EU€40,009 in 2007). These costs were determined from hospital records, questionnaires and estimated from expert opinion.^(33)^ Additionally, haematological cancers have been identified as some of the most expensive cancers to treat.^(56)^ Further research is needed to determine financial costs associated with use of the ND in a broader context including the financial impact on people following the ND.

Understanding the impact of dietary quality on the quality of life (QoL) among individuals with cancer is paramount. QoL when consuming an ND has only been assessed in populations of children and young people with cancer, which found following an ND was associated with a decreased QoL compared to the comparator diets.^(24,29)^ More research is needed as QoL is shown to be improved by eating with other people^(57)^ and this social connection remains critical – potentially more so – in people with cancer.^(58)^ Currently there is limited data on how the ND affects the way people eat with others. A qualitative study of older people with cancer^(57)^ found they experienced taste alterations and decreased appetite due to treatment and decreased social interactions around food but had increased family connection irrespective of food.^(57)^ As QoL is lower in people with cancer^(59,60)^ it is important that future studies consider the impact of ND on QoL in all populations.

A key finding was of the small number of studies that looked at diet acceptability and diet adherence there was no significant difference between the ND and comparator for acceptability^(10,24,30)^ however, adherence was significantly greater for the comparator diets.^(24,32)^ As these studies were conducted in 2006, 2012, and 2019, more recent studies may be needed to confirm these findings with contemporary menu designs.

Another major gap identified was how the ND impacts (i.e. procurement, cooking and safe handing, and storage) the normal routine of those with cancer, as well as their friends and family. None of the included studies reported on this area. While studies have considered what impact cancer treatment has had on families and lifestyle,^(61,62)^ none have assessed the impact of the ND.

A major strength of the present research is that, to our knowledge, this is the first scoping review of the ND for those with cancer, to scope the literature for all health-related outcomes using a systematic search process. The present scoping review is not without limitations. Samples sizes of the included studies – mainly of RCTs – were relatively small. Few studies assessed each included outcome making it challenging to draw conclusions from this. Due to variability of the ND, each study had a different definition in addition to the comparator diet used, however, most were a form of the food safety diet. Additionally, each study had implemented prophylactic measures in their study population in addition to the ND to reduce infection making it difficult to draw conclusions across the data. Included studies did not have consistent units or measures across outcomes particularly for the outcome of weight loss. It was difficult to determine whether there was a true difference in costs between the ND and the comparator due to ambiguity in the included study.

## Conclusion

Despite the need for further research into several areas related to cancer and the administration of the ND, the current evidence suggests that the ND does not serve its original purpose: to reduce the risk of infection in this population. Additionally, the ND may lead to malnutrition due to it lacking variety and providing an unpleasant experience at mealtimes, may be costing us more in the long-term, and has been shown to decrease paediatric patient quality of life. This is of particular importance as the ND is used for patients with cancer by more than 50% of hospitals in Europe and China.^(17–22)^ Further research is needed regarding how the ND affects the microbiome and quality of life of its consumers as well as associated costs, but in the interim, it is important for hospitals and other institutions providing an ND to their patients to liberalise the diet wherever possible.

## Abbreviations

**ND:** Neutropenic Diet; **QoL:** Quality of Life; **RCT:** Randomised Control Trial; **HSCT:** Haematopoietic Stem Cell Transplant; **ESPEN:** European Society for Clinical Nutrition and Metabolism; **MDR:** Multidrug Resistant; **GvHD:** Graft vs Host Disease.
